# The attitudes of people with sarcoma and their family towards genomics and incidental information arising from genetic research

**DOI:** 10.1186/2045-3329-3-11

**Published:** 2013-07-30

**Authors:** Mary-Anne Young, Amy Herlihy, Gillian Mitchell, David M Thomas, Mandy Ballinger, Kathy Tucker, Craig R Lewis, Susan Neuhaus, Jane Halliday

**Affiliations:** 1 Department of Oncology, University of Melbourne, Peter MacCallum Cancer Centre, Locked Bag 1, A’Beckett Street, Victoria 8006, Australia; 2 Public Health Genetics, Murdoch Childrens Research Institute, The Royal Children's Hospital, Flemington Road Parkville, Victoria 3052, Australia; 3 Hereditary Cancer Clinic, Prince of Wales Hospital, Prince of Wales Hospital, High St, Randwick 2031, Australia; 4 Dept of Medical Oncology, Prince of Wales Clinical School, University of New South Wales, Barker St, Randwick, NSW 2031, Australia; 5 Department of Surgery, University of Adelaide, Royal Adelaide Hospital, North Terrace, Adelaide, SA 5000, Australia; 6 Department of Paediatrics, University of Melbourne, Melbourne, VIC 3010, Australia

## Abstract

**Purpose:**

The study aimed to examine attitudes of individuals diagnosed with sarcoma and their family members towards genetics, genomic research and incidental information arising as a result of participating in genetic research.

**Methods:**

A questionnaire was administered to 1200 individuals from the International Sarcoma Kindred Study (ISKS). Respondents were divided into three groups: individuals affected with sarcoma (probands), their spouses and family members.

**Results:**

Approximately half of all research participants felt positively towards new discoveries in human genetics. Overall, more were positive in their attitudes towards genetic testing for inherited conditions (60%) but family members were less so. Older participants reported more highly positive attitudes more often than younger participants. Males were less likely to feel positive about new genetic discoveries and more likely to believe they could modify genetic risk by altering lifestyle factors. Almost all ISKS participants believed participants would like to be given ancillary information arising as a result of participating in genetic research.

**Conclusions:**

The only difference between the study groups was the decreased likelihood of family members being highly positive about genetic testing. This may be important if predictive testing for sarcoma becomes available. Generally ISKS research participants supported the notion of returning incidental genetic information to research participants.

## Introduction

The evolution of new genomic technologies such as chromosomal microarray analysis and massively parallel sequencing of targeted regions and potentially the whole exome or genome, can result in the identification of incidental genetic information, in both clinical practice and medical research. This information has potential relevance to individuals and their families. Extensive discussion amongst researchers, health professionals, lawyers and bioethicists continues in an attempt to resolve the myriad of dilemmas including balancing the medical, ethical, psychosocial and social implications of new genomic technologies [1-4]

Reaching consensus about the innumerable complexities surrounding this issue creates enormous challenges. In the research setting the issues include considering the appropriateness of returning certain findings; how to obtain the funding required to return results so that the research is not crippled; balanced against avoiding undue distress to research participants and appropriate clinical follow up. The culmination of a two year project funded by the National Institutes of Health has resulted in published recommendations to assist biobanks and archived data sets in managing incidental information and genomic research results [5,6].

It is equally important that the discussion include the contribution and opinions of research participants and the general public. There is mounting evidence showing they are interested in receiving incidental information [7-11]. Previous research has shown that individuals’ knowledge and attitudes are associated with their response to genetic information, although greater knowledge does not lead to unequivocal acceptance of genetic technology and testing [12-16].

Sarcomas are malignant tumours of connective tissue. They are rare although contribute disproportionately to cancer burden as they often affect the young, treatment is costly and prolonged, and morbidity and mortality high [17,18]. Genetic factors appear important in sarcoma, although have not been well studied. The ISKS, a clinic-based study, recruits individuals with sarcoma agnostic to family history, as well as their partners and genetic relatives creating a resource for future research into the genetic factors contributing to the hereditary risk of developing sarcoma [19,20].

We sought to provide additional data to the discussion about incidental information by conducting a study with individuals affected by adult-onset sarcoma (probands), their spouses and family members, all of whom were participants in the ISKS.

Our aim in this exploratory study was to examine and compare the attitudes towards and opinions about genomic research and incidental information between these three groups.

## Materials and methods

### Source of participants

Individuals with a histologically confirmed sarcoma were recruited through key sarcoma clinics throughout Australia. If the proband was diagnosed <45 yrs or was diagnosed ≥45 yrs and had a significant family history of cancer, genetic relatives were additionally recruited. Age matched (±5 yrs) comparison groups were also recruited. All participants were asked to complete a broad baseline questionnaire and provide biospecimens.

### Survey development

We developed a discrete set of questions to include in the larger baseline questionnaire administered to all study participants. The questions on attitudes to genetic testing and genomic research were developed using the UK Human Genetics Commission 2001 report into Public Attitudes to Human Genetic Information [21] and study specific questions were designed to examine attitudes to incidental information arising from participation in research.

The questions asked about:

1. Beliefs in the genetic versus environmental contribution to aspects of health and well-being.

2. Feelings towards new genetic discoveries.

3. Attitudes towards genetic testing for inherited conditions.

4. Attitudes towards the possibility of “incidental findings” as a result of participating in genetic research.

Questions about new genetic discoveries covered six items on a seven point Likert scale (bored/excited, valuable/worthless, uninterested/interested, indifferent/passionate, don’t care/care and important/unimportant). Scores for the six items were averaged, and participants were categorised as being ‘highly positive’ (mean ≥ 6) or ‘less positive’ (≤ 6).

The questions about genetic testing for inherited conditions covered five domains on a seven point Likert scale (favourable/unfavourable, calm/anxious, trusting/sceptical, good idea/bad idea and acceptable/unacceptable) with participants categorised as being ‘highly positive’ or ‘less positive’ on the basis of their mean scores as above.

The study received ethical approval from the Human Research Ethics Committee at the main study site, Peter MacCallum Cancer Centre, Victoria, Australia as well as all seven other recruiting sites throughout Australia.

### Data collection

Data were collected from July 2009 to May 2012. The questionnaire relating to this study was self-administered at the time of recruitment to ISKS.

### Data analysis

The data were analysed in STATA (version 12.1), with the participant category (proband, family member or spouse/partner) being the main exposure of interest. The distributions of all covariates and outcomes were examined using Chi square tests for heterogeneity for normally distributed, categorical data. Multivariable logistic regression (for binary outcomes) enabled adjustment of the outcomes of interest for potential confounders or partial intermediates described as explanatory factors. These included age, gender, education, and country of birth.

Clustering within families was accounted for by fitting Generalised Estimating Equation regression models.

## Results

Table 1 shows the participant characteristics in terms of age, gender, relationship status, education and country of birth.

**Table 1:** Participant characteristics

	**All**	**Proband**	**Family**	**Spouse**	**p-value**
	**n=1200**	**n=524**	**n=514**	**n=162**	
**Age at study**					0.25
18 to 39 years, n (%)	332 (28%)	159 (30%)	137 (27%)	36 (22%)	
40 to 60 years, n (%)	472 (39%)	192 (37%)	210 (41%)	70 (43%)	
61 years & older, n (%)	396 (33%)	173 (33%)	167 (32%)	56 (35%)	
**Female, n (%)**	638 (53%)	241 (46%)	303 (59%)	94 (58%)	
**Partnered, n (%)**	859 (72%)	343 (66%)	362 (71%)	-	
**Highest education completed**					0.08
High School, n (%)	268 (22%)	110 (21%)	129 (25%)	29 (18%)	
University/Vocational, n (%)	603 (50%)	260 (50%)	262 (51%)	81 (50%)	
**Country of birth**					0.005
Australia, n (%)	921 (77%)	382 (73%)	418 (81%)	121 (75%)	
**Cancer**					<0.001
Yes, n (%)	624 (52%)	-	96 (19%)	4 (2%)	

Forty four percent of the total sample was adult probands i.e. with sarcoma, with nineteen percent having had two or more primary malignancies. Fifty percent had been diagnosed with sarcoma within 12 months of participating in the study.

There were no marked differences between the three categories of participants in age group frequencies, relationship status or education, but there were differences in terms of gender (Chi square = 18.97, p = <0.001) with almost 60% of spouses and family members being female and less than 50% of the probands female. There were also differences in terms of country of birth (COB), with 73% of probands born in Australia compared to 81% of family members (Chi square = 10.76, p < 0.005).

### Beliefs in the genetic versus environmental contribution to aspects of health and well-being

**Figure 1:**
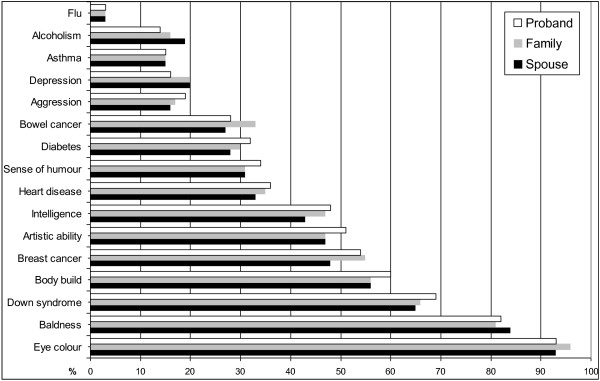
Proportion of probands, family members and spouses who indicated characteristic was inherited.

There were no significant differences observed between probands, spouses and family members in beliefs in an inherited contribution to the characteristics or health condition (Figure 1). A greater number of participants in all groups assigned greater emphasis to the genetic contribution to breast cancer as compared to bowel cancer.

### Feelings about genetic discoveries

The proportion of probands, family members and spouses that felt highly positive about new genetic discoveries was 53%, 49.2% and 57.4% respectively. After adjusting for age, gender, education, country of birth and genetic beliefs, there was no difference in the odds of a family member or spouse having different feelings from the proband (Table 2). Older participants (≥61 years), were more likely to feel highly positive about genetic discoveries when compared with younger participants (AdjOR 1.47, 95% CI: (1.06-2.05)) and males had a lower odds of feeling highly positive compared to females (AdjOR 0.66, 95% CI: (0.52-0.82)). Individuals who had a university education had an increased odds of feeling highly positive compared to those of lower education (AdjOR 1.45, 95% CI: (1.07-1.97)).

**Table 2:** Feelings about genetic discoveries

		**Feelings about genetic discoveries**					
		**Bivariable analysis**			**Multivariable analysis**		
**Variable**	**Category**	**OR**	**95% CI**	**p**	**Adj OR**	**95% CI**	**p**
Participant	Proband	Ref	Ref	Ref	Ref	Ref	Ref
	Family member	0.86	0.67-1.09	0.22	0.80	0.62-1.05	0.11
	Spouse	1.19	0.84-1.70	0.33	1.15	0.81-1.63	0.45
Age at study	18 to 39 years	Ref	Ref	Ref	Ref	Ref	Ref
	40 to 60 years	1.07	0.81-1.42	0.63	1.04	0.77-1.41	0.79
	61 years and older	1.41	1.05-1.89	**0.02**	1.47	1.06-2.05	**0.02**
Gender	Female	Ref	Ref	Ref	Ref	Ref	Ref
	Male	0.67	0.54-0.84	**0.00**	0.66	0.52-0.82	**<0.001**
Highest education	Primary School	Ref	Ref	Ref	Ref	Ref	Ref
	High School	1.016	0.74-1.40	0.92	1.20	0.85-1.68	0.30
	University/Vocational	1.311	1.00-1.72	**0.05**	1.45	1.07-1.97	**0.02**
Country of birth	Australia/NZ	Ref	Ref	Ref	Ref	Ref	Ref
	Other	1.350	1.03-1.77	**0.03**	1.21	0.89-1.65	0.22

Logistic regression analysis. 0 = Less positive, 1 = Highly positive.

Multivariable analysis adjusted for age, gender, education, COB, genetic beliefs.

### Attitudes towards genetic testing

The proportion of probands, family members and spouses that felt highly positive about genetic testing for inherited conditions was 66.8%, 59.7% and 61.7% respectively. All groups were asked about their attitudes to genetic testing for inherited conditions (in general). Compared to probands, family members had a lower odds of being highly positive (AdjOR 0.69, 95% CI: (0.53-0.88)) (Table 3). Older participants (≥61 yrs) reported significantly higher positive attitudes towards genetic testing than younger participants (AdjOR 2.06, 95% CI:(1.50-2.84)).

**Table 3:** Attitudes towards genetic testing

		**Attitudes towards genetic testing**					
		**Bivariable analysis**			**Multivariable analysis**		
**Variable**	**Category**	**OR**	**95% CI**	**p**	**Adj OR**	**95% CI**	**p**
Participant	Proband	Ref	Ref	Ref	Ref	Ref	Ref
	Family member	**0.74**	**0.57-0.95**	**0.02**	0.69	0.53-0.88	**<0.003**
	Spouse	0.80	0.56-1.16	0.24	0.78	0.54-1.12	0.18
Age at study	18 to 39 years	Ref	Ref	Ref	Ref	Ref	Ref
	40 to 60 years	1.15	0.86-1.53	0.34	1.22	0.91-1.65	0.19
	61 years and older	**1.83**	**1.34-2.48**	**<0.001**	2.06	1.50-2.84	**<0.001**
Gender	Female	Ref	Ref	Ref	Ref	Ref	Ref
	Male	0.85	0.67-1.08	0.18	0.82	0.64-1.04	0.10
Highest education	Primary School	Ref	Ref	Ref	Ref	Ref	Ref
	High School	1.01	0.73-1.15	0.94	1.32	0.92-1.91	0.14
	University/Vocational	0.99	0.75-1.31	0.94	1.22	0.88-1.68	0.23
Country of birth	Australia/NZ	Ref	Ref	Ref	Ref	Ref	Ref
	Other	0.87	0.66-1.15	0.32	0.75	0.55-1.03	0.07

Logistic regression analysis. 0 = Less positive, 1 = Highly positive.

Multivariable analysis adjusted for age, gender, education, COB, genetic beliefs.

### Beliefs about the ability to modify inherited risk by lifestyle factors

Forty percent of probands, 44% of spouses and 38% of family members believed it was possible to alter an inherited risk by modifying lifestyle factors e.g. eating better, having more exercise or following a special health program (Table 4). There were no differences in participant category although males were more likely to believe risk could be altered (AdjOR 1.64, 95% CI: (1.28-2.08)) as were individuals who had tertiary education (AdjOR 1.39, 95% CI: (1.01-1.91)).

**Table 4:** Beliefs about altering inherited risk by modifying lifestyle factors

		**Beliefs about altering disease risk**					
		**Bivariable analysis**			**Multivariable analysis**		
**Variable**	**Category**	**OR**	**95% CI**	**p**	**Adj OR**	**95% CI**	**p**
Participant	Proband	Ref	Ref	Ref	Ref	Ref	Ref
	Family member	0.91	0.71-1.17	0.47	0.98	0.74-1.30	0.85
	Spouse	1.19	0.84-1.70	0.33	1.29	0.90-1.84	0.17
Age at study	18 to 39 years	Ref	Ref	Ref	Ref	Ref	Ref
	40 to 60 years	0.93	0.70-1.24	0.64	0.95	0.72-1.27	0.75
	61 years and older	0.74	0.55-1.00	0.05	0.78	0.56-1.08	0.14
Gender	Female	Ref	Ref	Ref	Ref	Ref	Ref
	Male	1.61	1.28-2.04	**<0.001**	1.64	1.28-2.08	**<0.001**
Highest education	Primary School	Ref	Ref	Ref	Ref	Ref	Ref
	High School	1.29	0.92-1.81	0.13	1.21	0.84-1.73	0.30
	University/Vocational	1.46	1.11-1.94	**0.01**	1.39	1.01-1.91	**0.04**
Country of birth	Australia/NZ	Ref	Ref	Ref	Ref	Ref	Ref
	Other	1.15	0.88-1.51	0.32	1.15	0.83-1.59	0.41

Logistic regression analysis. 0 = Not possible to alter risk, 1 = Possible to alter risk.

Multivariable analysis adjusted for age, gender, education, COB, genetic beliefs.

### Opinions and attitudes towards incidental information arising as a result of research

The questionnaire used four hypothetical scenarios to determine what conditions people would like to be informed about when incidental information arises as a result of participating in research. These scenarios were (1) risk for a disease caused by a single gene for which there is no prevention e.g. inherited blindness; (2) risk for a disease caused by one gene for which there is evidence that prevention such as screening or treatment can change the risk e.g. breast or bowel cancer; (3) risk for a disease caused by many genes which can have a major impact on health for which there is specified treatment or lifestyle modification such as improving diet, stopping smoking, or increasing exercise which can modify the risk e.g. heart disease; (4) risk for a disease caused by many genes which usually have a lower impact on health for which there is treatment as well as lifestyle changes which can modify the risk e.g. asthma.

Table 5 shows that almost all ISKS participants responded saying they believed that people taking part in genetic research would like to be informed about incidental findings for which there is prevention and/or treatment. Overall, fewer participants felt people would like to be informed about a single gene disorder for which there is no prevention with a higher proportion of spouses (76%) than probands (67%) and family members (66%) responding positively to this. Adjusting for age, gender, education and country of birth did not alter this significance (AdjOR 1.52, 95% CI:(1.02-2.26)).

**Table 5:** Participant opinions and attitudes towards genetic testing and research: proportion of each participant group responding to the different scenarios

**When people take part in genetic research, I think they would like to be informed about:**	**All**	**Proband**	**Family**	**Spouse**	**P-value**
	**n=1200**	**n=524**	**n=514**	**n=162**	
Known genetic conditions caused by one gene, for which there is no prevention	68%	67%	66%	76%	**0.05**
Known genetic conditions caused by one gene, for which there is prevention or treatment that can change the risk	94%	94%	93%	95%	0.43
Known genetic conditions caused by many genes, which can have a major impact on health, for which there is treatment as well as lifestyle factors which can modify the risk	93%	93%	93%	96%	0.70
Known genetic conditions caused by many genes, which usually have a lower impact on health, for which there is treatment as well as lifestyle factors which can modify the risk	90%	89%	89%	92%	0.83

## Discussion

This study about ISKS participants’ attitudes towards genomics and incidental information generated through research brings the consumer viewpoint to the discussion about the numerous complex issues raised by the use of new genetic technology. Our results showed that approximately half of the ISKS participants, including individuals affected with sarcoma, their spouses and family members felt positively towards new discoveries in human genetics. Their attitudes towards genetic testing for inherited conditions were slightly more positive. We did not measure knowledge, although previous research has shown an association between knowledge about genes and attitudes towards genetic tests with higher levels of knowledge being positively associated with acceptance of biotechnology, attitudes to science and attitudes towards genetic testing [12,22,23]. ISKS participants, though not necessarily reflective of the general population, are representative of those involved in disease associated research.

As shown in Figure 1 there was no significant difference between the groups concerning their beliefs about the genetic contribution to aspects of health and well-being (see Figure 1). Rather, there was remarkable consistency amongst the three groups. Of interest was the number of participants who assigned greater emphasis to the genetic contribution to breast cancer as compared to bowel cancer despite the heritable component of bowel cancer being up to 30% [24]. This highlights the need for continuing education to improve the public’s knowledge and genetic literacy about genetics and genetic testing. The best possible use of genetic information to improve health depends on this. Education should include information about the social and ethical issues associated with the development of genomic technology and genetic testing. This will ensure those faced with the choice of accessing and utilising genetic information have the necessary background knowledge for informed decision making. Moreover, it will mean the public can meaningfully contribute to the debate about emerging genetic technologies, cognisant about all the issues. Education must also be inclusive of health professionals, particularly in primary care, as they will be increasingly likely to play a role in the delivery of complex genetic information to patients.

### Link between knowledge and attitudes to genetics

It has been suggested that better genetic knowledge results in a greater appreciation and support of science. Accordingly, a lack of knowledge may explain the reservations and fears some of the public express when asked about genetic technologies [25] and the findings in this study that only half felt positively about new genetic discoveries. Whilst there is debate about the role of knowledge and its relationship to the acceptance of gene technology some studies have identified knowledge as a factor possibly influencing the public’s attitudes and hence their support to science, biotechnology and genetics, which can be either positive or negative [26].

Knowledge is comprised of objective knowledge (i.e. what people actually know), and subjective knowledge (i.e. what people perceive they know). Objective knowledge is derived from diverse sources and may not always be correct. For example, films and books with important genetic themes have often been critiqued due to their inaccuracy or for distortion of the scientific facts [27] although their influence cannot be underestimated in influencing the general public’s understanding and perception of genetics.

The relationship between knowledge and attitudes is not clear and other factors like emotions and previous experience with genetic disease have also been shown to influence perception. This is known as experiential knowledge [25,26]. A recent study examining factors that influence people’s acceptance of gene technology showed that the experiential system which is based on affect, narratives, associations and imageries is more influential in lay people’s perceptions of genetic technology than the analytical system which is based on rational interpretation and evidence [26]. Experiential knowledge may explain the finding that probands in the ISKS cohort were more positive in their attitudes towards genetic testing than family members (see Table 3). Probands have been diagnosed and treated for sarcoma, thus could be considered to have intimate experiential knowledge. They may also have had some prior knowledge about the role of genetics in sarcoma through discussion with their treating doctors and/or as part of the consent process to the study. As well, they may believe they could have personally benefitted from genetic information in regards to earlier diagnosis and personalised medical treatment if it existed. This raises the tantalising nature of genetic information and the public’s tendency to overrate its power to predict and/or prevent disease [28].

Individuals with higher levels of education have been shown to have more positive attitudes towards genetic technology [23,26,29]. Higher education is theoretically associated with greater knowledge and it is possible that this group may be more accepting of genetic technology. Education was a significant factor that influenced ISKS participant’s feelings about genetic discoveries and beliefs about the ability to modify inherited risk. Individuals who had a university or vocational education were more likely to feel positive about new genetic discoveries (Table 3) and were more likely to believe in the ability to modify genetic risk (Table 4).

### Influence of sociodemographic variables

As stated above, education was an important confounder in this study as was age, particularly how participants felt about new genetic discoveries and attitudes towards genetic testing with older participants reporting higher positive attitudes as compared to younger participants (Table 3). Our finding could be explained by the fact that older people in our study may have had greater concern about the implications to their own offspring and therefore feel more hopeful about new medical developments, including genetics. This finding is different to a population based Finnish study where older participants were more likely to state they ‘did not know’ when asked about their attitudes towards genetic testing [12].

Males in the ISKS cohort were less likely to feel positive about new genetic discoveries and their scores suggested a stronger belief that inherited risk could be altered by modifying lifestyle factors when compared to females. This finding about males feeling less positive is in contrast with previous research which has shown that men tend to assess genetic technology more positively than women [26]. The higher expression of this belief by males in the ISKS cohort could be explained by an active coping style or male socialisation patterns [30]. Men are generally not active health seekers although as they have a more active coping style they want to do something [31]. These finding must be considered in the light of previous research which has demonstrated that intentions or attitudes may not always predict behaviour [32]. In addition, although individuals state they wish to use genetic information to modify risk, studies of direct to consumer genetic testing have so far shown it does not lead to changes in behaviour relating to exercise and diet, or lead to further screening and preventative action [33,34].

### Incidental findings

The majority of ISKS participants thought research participants would like to be informed about incidental findings arising as a result of participating in medical research. This finding supports mounting evidence in the literature that individuals are interested in receiving incidental information for reasons not only related to clinical utility but also personal utility, autonomy and choice. This includes gaining a sense of personal control and helping to plan for the future [7,9,10,35,36].

A novel finding of this study is that less ISKS participants thought people would like to be informed about monogenic conditions where there is no prevention as compared to monogenic conditions where there is existing prevention or treatment or polygenic disorders. Importantly, there are few proven risk modification strategies for the most commonly recognised sarcoma predisposition syndrome, Li-Fraumeni syndrome (LFS). The lifetime risk for cancer in classic LFS approaches 100% for females [37]. The desire to know and not know about unmodifiable risk is therefore important to further research into the genetic causes of sarcomas, and supports the notion of individual choice when making decisions about what findings participants receive. Lay individuals, in a recent study exploring attitudes about the disclosure of incidental information, expressed the importance of pre-test discussions to establish participant views around the type of information they would like to receive and avoid unanticipated results. They also stated they did not want health professionals to make decisions on their behalf [35]. The results from our study suggest that the public can be discerning when presented with choices around the type of information they wish to receive or not receive.

Information participants choose to receive may not be the same as what clinicians consider is important to return. The National Institutes of Health [6] use the term ‘actionability’ to assist in determining whether or not to return information. They define actionability from the perspective of the individual facing risk and potential disease. Actionability can mean different things to different parties; including researchers, clinicians, human research ethics committees, research participants and the public. Whilst clinicians might consider clinical utility and ability to interpret and communicate such information, some research participants find genetic risk information valuable for reasons including personal utility [8,38]. Therefore, it is essential research participants are involved in the decision about what information they wish to receive when they consent to participate in research. A recent study found that 75% of potential research participants stated they would be less likely to participate in research if incidental findings were not returned indicating some participants may see returning information as a proviso of enrolment into research [39].

The strength of this study is the large sample size and the broad representation from multiple clinics across Australia. A limitation to this research is that the questions were general and knowledge was not assessed. Therefore the association between knowledge and attitudes towards incidental findings is not able to be assessed here and is worthwhile exploring in further research.

A further limitation is that this research is based on hypothetical scenarios and responses from individuals when presented with a hypothetical scenario are not always consistent with future individual behaviour [32].

## Conclusion

In conclusion, we have shown that at least half of ISKS participants, no matter whether they were individuals affected with cancer or their family members, were positive about new genetic discoveries and genetic testing. Age and gender were factors that influenced how people thought about genetic discoveries and genetic testing. Although intention to receive results may not translate into action, we believe that if genetic testing for sarcoma becomes available in the foreseeable future, it is likely that family members may demonstrate more reservation towards such testing than probands and their spouses and this should be taken into consideration. Finally, the majority of ISKS participants believe people would like to be informed about incidental information arising as a result of research.

## Competing interests

The authors declare that they have no competing interests.

## Authors’ contributions

MAY & JH designed the study. MAY, JH and AH analysed the data. AH prepared the results for the manuscript. MAY and JH drafted the manuscript. AH, GM, DT, MB, KT, CL and SN all contributed equally to the preparation of the manuscript. All authors read and approved the final manuscript.
